# Genomewide evidence of environmentally mediated secondary contact of European green crab (*Carcinus maenas*) lineages in eastern North America

**DOI:** 10.1111/eva.12601

**Published:** 2018-02-23

**Authors:** Nicholas W. Jeffery, Ian R. Bradbury, Ryan R. E. Stanley, Brendan F. Wringe, Mallory Van Wyngaarden, J. Ben Lowen, Cynthia H. McKenzie, Kyle Matheson, Philip S. Sargent, Claudio DiBacco

**Affiliations:** ^1^ Fisheries and Oceans Canada Northwest Atlantic Fisheries Centre St. John's NL Canada; ^2^ Faculty of Computer Science Dalhousie University Halifax NS Canada; ^3^ Department of Ocean Sciences Memorial University of Newfoundland St. John’s NL Canada; ^4^ Fisheries and Oceans Canada Bedford Institute of Oceanography Dartmouth NS Canada

**Keywords:** *Carcinus maenas*, European green crab, invasive species, RAD‐seq, seascape genetics

## Abstract

Genetic‐environment associations are increasingly revealed through population genomic data and can occur through a number of processes, including secondary contact, divergent natural selection, or isolation by distance. Here, we investigate the influence of the environment, including seasonal temperature and salinity, on the population structure of the invasive European green crab (*Carcinus maenas*) in eastern North America. Green crab populations in eastern North America are associated with two independent invasions, previously shown to consist of distinct northern and southern ecotypes, with a contact zone in southern Nova Scotia, Canada. Using a RAD‐seq panel of 9,137 genomewide SNPs, we detected 41 SNPs (0.49%) whose allele frequencies were highly correlated with environmental data. A principal components analysis of 25 environmental variables differentiated populations into northern, southern, and admixed sites in concordance with the observed genomic spatial structure. Furthermore, a spatial principal components analysis conducted on genomic and geographic data revealed a high degree of global structure (*p* < .0001) partitioning a northern and southern ecotype. Redundancy and partial redundancy analyses revealed that among the environmental variables tested, winter sea surface temperature had the strongest association with spatial structuring, suggesting that it is an important factor defining range and expansion limits of each ecotype. Understanding environmental thresholds associated with intraspecific diversity will facilitate the ability to manage current and predict future distributions of this aquatic invasive species.

## 1 INTRODUCTION

The contemporary study of “seascape genetics” (Riginos & Liggins, 2013) is increasingly revealing fine‐scale local structuring through the use of genomic data, allowing robust investigations of the forces influencing spatial genetic variation. Fine‐scale population structure in marine species (Benestan et al., 2015; Bradbury et al., 2010; Harrisson et al., 2017) may be driven by a number of processes, including secondary contact among lineages or closely related species (e.g., Abbott et al., 2013; Hewitt, 1996, 2000; Mayr, 1942), recent adaptation along an environmental gradient (e.g., Endler, 1977; Sanford & Kelly, 2011), or some combination of physical isolation, genetic drift, and selection (Harrison & Larson, 2016). In practice, these processes may be difficult to distinguish, particularly in nonequilibrium systems (Bierne, Welch, Loire, Bonhomme, & David, 2011; Harrisson et al., 2017). Nonetheless, secondary contact has a long history of identification as a primary driver of clinal variation, and is increasingly implicated as a cause of fine geographic scale variation, and a source of heterogeneous genomic divergence in marine species (Barney, Munkholm, Walt, & Palumbi, 2017; Berg et al., 2017).

The advent of genomewide association studies, through technologies such as restriction‐site associated DNA sequencing (RAD‐seq), transcriptomics, and whole genome sequencing provides a novel platform to evaluate genomic variation and divergence associated with allopatric isolation or selection (Benestan et al., 2015; Sherman et al., 2016; Tepolt, 2015; Tepolt & Palumbi, 2015). Although the application of RAD‐seq to evaluate genetic variation has recently come under fire (i.e., Hoban et al., 2016; Lowry et al., 2017; but see McKinney, Larson, Seeb, & Seeb, 2017), owing to the sparsity of markers across the genome (hundreds to tens of thousands of markers across genomes which are hundreds of millions to billions of base pairs long), it has been proven as a capable tool to evaluate population structure and environmental associations, provided careful interpretation and acknowledgment of the limitations (Bierne, 2010; Bierne et al., 2011). Fine‐scale population structure has been identified in a variety of taxa including kelp, pelagic fish, mollusks, echinoderms, and crustaceans (see Riginos & Liggins, 2013 and references therein) using high‐throughput sequencing at a range of patch sizes spanning several meters to thousands of kilometers. Invasive species where hybridization occurs among invasive and native species or waves of invasion represent an unique natural history experiment (e.g., Fitzpatrick et al., 2010; Saarman & Pogson, 2015; Viard, David, & Darling, 2016) and allow the examination of both how secondary contact may influence the distribution of genomic variation as well as how hybridization and introgression maybe be influenced by factors such as environmental variation.

The European green crab (*Carcinus maenas*) is a poignant example of such an invasive species. Green crab are highly tolerant of a wide range of temperatures and salinities, encompassing a 25°C range in sea surface temperatures in their native range and a range in salinity tolerance of 4–52‰ in adults (Klassen & Locke, 2007; Tepolt & Somero, 2014). At least two independent introductions of green crab have occurred since the 1800s (Audet et al., 2003; Blakeslee et al., 2010; Roman, 2006), resulting in the establishment of genetically distinct northern and southern ecotypes (Jeffery, DiBacco, Van Wyngaarden et al., 2017; Tepolt & Palumbi, 2015). These ecotypes correspond to northern and southern populations in the native range (Roman, 2006). Audet et al. (2003) revealed that prior to the introduction of the presumably cold‐adapted northern European green crab lineage, cold coastal currents appear to have halted the northward expansion of the southern green crab ecotype at approximately the latitude of Halifax, NS (~44.6°N). Similarly, cold winters in the Gulf of Maine have previously been linked to increased adult mortality whereas warm winters have been linked to increased recruitment (Yamada & Kosro, 2010). In both the native and introduced ranges, the mean high temperature at which cardiac function fails in adult crabs is consistently higher in southern populations which are adapted to overall warmer sea surface temperatures (Tepolt & Somero, 2014). This latitudinal pattern in thermal tolerance likely evolved in the native range and has since mediated the success and expansion of populations in eastern North America (Tepolt & Somero, 2014). It is clear that the environment plays an important role in the population structure and distribution of this species, though the specific nature of this relationship remains unknown.

Here, we investigate environmental correlates of secondary contact in this species using genomewide climate‐associated SNPs obtained by RAD sequencing of green crabs from locations along their invasive range in eastern North America. We first seek to explore the presence of genomic–environmental‐associated variation using latent factor mixed models (LFMM; Frichot, Schoville, Bouchard, & François, 2013) and a Bayesian genome scan method (Villemereuil & Gaggiotti, 2015). Based on physiological evidence from Tepolt and Somero (2014), we predict that seasonal temperature minima are limiting the range expansion of the invasive green crab ecotypes and that environmentally associated genomic regions will track a steep cline in temperature previously reported in Atlantic Canada. This work builds directly on previous studies highlighting the genomic differences (Jeffery, DiBacco, Van Wyngaarden et al., 2017; Tepolt & Palumbi, 2015) and subsequent hybridization of two independent waves of invasion (Darling, Tsai, Blakeslee, & Roman, 2014; Jeffery, DiBacco, Wringe et al., 2017) to better understand the environmental correlates of population specific invasion success and secondary contact in this species. Our results will provide insight into how secondary contact dynamics can be correlated with environmental heterogeneity potentially constraining the success of coastal marine invaders, and will help to understand and predict future range expansions and contractions of green crab ecotypes.

## 2 MATERIALS AND METHODS

### 2.1 Environmental predictors

We selected 25 environmental variables, specifically annual and seasonally averaged bottom and surface temperatures and salinities, which have been noted as ecologically relevant to *C. maenas* (Klassen & Locke, 2007). A decadal average (2002–2012) was taken for each of four three‐month seasonal aggregations corresponding with winter (January–March), spring (April–June), summer (July–September), and fall (October–December). Seasonal sea surface temperature (SST) and sea surface salinity (SSS) were assembled at a 1 km^2^ resolution from Level 3 SST climatological satellite data (Aqua‐MODIS 4‐micron nighttime) (Sbrocco & Barber, 2013) and global oceanographic climatological SSS composites (Tyberghein et al., 2012). All climatological data layers were converted to ASCII grid with WGS84 global stereographic projection and a uniform land mask applied. Environmental variables (SST and SSS) corresponding to each geo‐referenced sample location were subsequently extracted.

### 2.2 Detection of environment associated loci

**Figure 1:**
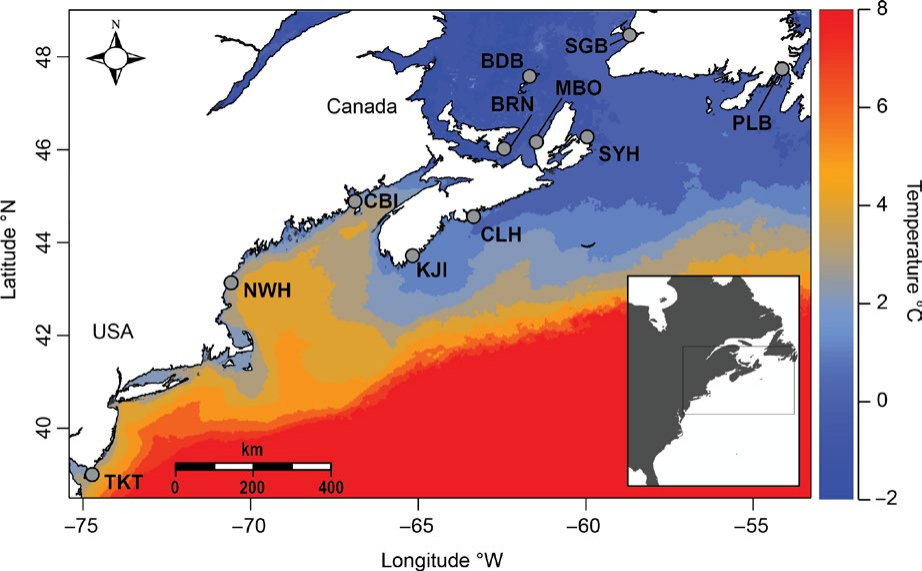
Map of eastern North America, including the 11 sampling locations where green crabs (*Carcinus maenas*) were collected. The Atlantic Ocean winter sea surface temperature (°C) is shown across this range

We used previously published RAD data from Jeffery, DiBacco, Van Wyngaarden et al. (2017) which included 9,137 SNPs in 241 individual crabs from 11 sites ranging from New Jersey, USA, to southern Newfoundland, Canada (Figure 1). To detect loci which show a strong association with the environmental data, we implemented latent factor mixed models (LFMM; Frichot et al., 2013) available in the R package *LEA* (Frichot & François, 2015) as the first method of outlier detection. This method detects correlations between environmental variables and genotypic variation to reveal outliers, while controlling for population structure (*K*) and unmeasured environmental variables as latent factors. We set *K* = 2 based on the results of previous discriminant analyses of principal components (DAPC) and STRUCTURE (Pritchard, Stephens, & Donnelly, 2000) results (Jeffery, DiBacco, Van Wyngaarden et al., 2017; Jeffery, DiBacco, Wringe et al., 2017). LFMM was run with a burn‐in of 5,000 iterations and 15,000 Markov Chain Monte Carlo (MCMC) iterations which were replicated five times. *Z*‐scores per locus were combined from the five replicates, and false discovery rates were evaluated using Benjamini and Hochberg (1995) adjusted *p*‐values. We then combined the lists of loci detected as outliers for each of the 25 environmental variables we tested, retaining all loci with an adjusted *p*‐value < .05.

We compared our results from LFMM to BayeScEnv (Villemereuil & Gaggiotti, 2015), an *F*_ST_‐based genome scan approach which accounts for environmental differentiation among populations. This method has a lower false discovery rate relative to purely *F*_ST_‐based methods (Villemereuil & Gaggiotti, 2015). We standardized the environmental variables for each population by taking the absolute difference from the mean and dividing each by the standard deviation as recommended by the program authors. We used 20 pilot runs with 50,000 MCMC iterations each and an initial burn‐in of 50,000 iterations for each of the 25 environmental parameters. The prior probability that a locus is an outlier was set to 0.1 to minimize the false discovery rate. As with LFMM, we combined all outliers for all environmental parameters and then compared the number of overlapping loci between the two methods. Outliers were subset from our full SNP panel using the R package *genepopedit* (Stanley, Jeffery, Wringe, DiBacco, & Bradbury, 2017) to generate our outlier panel of SNPs. Linkage disequilibrium *r* ^2^ values were calculated among all environmental outliers in PLINK (Purcell et al., 2007). Our nonoutlier panel of SNPs was then comprised of all SNPs not detected as outliers by either LFMM or BayScEnv, thus excluding any loci that were strongly correlated with the environment.

### 2.3 Environmental association and redundancy analysis

To determine the extent of global and local population structure of green crabs, we conducted a spatial principal component analysis (SPCA). We used the R function *CartDist* (Stanley & Jeffery, 2017) to first calculate least‐cost distances among populations in the R package *marmap* (Pante & Simon‐Bouhet, 2013) using land as an impenetrable barrier. We then used a nonmetric multidimensional scaling function in *vegan* (Oksanen, Kindt, Legendre, O'Hara, & Stevens, 2017) to convert these least‐cost distances into Cartesian coordinates which accounted for land barriers in the SPCA. The SPCA was run on the nonoutlier and outlier RAD panels, using the Delaunay triangulation method (Delaunay, 1934) in the R package *adegenet* (Jombart, 2008). We used Monte Carlo randomization tests with 1,000 permutations to assess the significance of global (high autocorrelation) and local (dissimilar neighboring sites) structures. We then extracted the first axis lagged scores from the environmental outlier SPCA and used a multiple linear regression to determine any significantly associated environmental variables, calculating the variance inflation factors (VIF) for each model and removing each variable with the highest VIF until all VIFs were <5, indicating the removal of collinearity among predictors. We additionally ran a logistic generalized linear model of the SPCA scores and latitude to model the observed cline in population structure. To verify that the VIF model selection worked, we additionally conducted elastic net regression on the SPCA lagged scores and environmental data which accounts for collinearity among predictors in the R package *glmnet* (Friedman, Hastie, & Tibshirani, 2010).

Next, we used a principal components analysis (PCA) in *adegenet* to evaluate environmental similarity among our sample locations using all environmental variables. We then used redundancy analyses (RDA) and partial redundancy analyses (pRDA) to account for the geographic autocorrelation of each site for both the outlier and nonoutlier locus allele frequencies using *vegan* to select which environmental variables best explain the genetic structure. Analyses of variance (ANOVA with 1,000 permutations) were used to assess the significance of each environmental parameter within the RDA. The *ordistep* function, a stepwise permutational ordination method, was used with 1,000 permutations to evaluate the environmental variables and create a model which best explained the spatial distribution of our genotype data.

Finally, we compared a simple isolation‐by‐distance (IBD) relationship as a null hypothesis to a genetic‐environment association controlling for geographic distances using multiple regression on distance matrices (MRM; Lichstein, 2007). For the IBD relationship, we used pairwise population linearized *F*_ST_ values as the response value and least‐cost distances calculated in *marmap* as the explanatory variable using the MRM function with 10,000 permutations in the *ecodist* R package (Goslee & Urban, 2007). We then conducted an MRM using the best‐supported environmental variable(s) and geographic distance from the above analyses to determine the significance of the environment on gene flow among populations.

### 2.4 Cline width and dispersal estimation

We used the R package *HZAR* (Derryberry, Derryberry, Maley, & Brumfield, 2014) on our 41 environmental outliers and 100 randomly sampled loci from the neutral SNP panel to model clines of allele frequency and least‐cost distances among sampling sites. Placentia Bay individuals were removed due to their anthropogenic introduction to the northern end of the sampling locations which would skew the cline width, as they are a known hybrid population (Blakeslee et al., 2010; Jeffery, DiBacco, Van Wyngaarden et al., 2017; Tepolt & Palumbi, 2015). We tested seven clinal models per locus, including one null model and combinations of fixed, free, or no scaling of maximum and minimum allele frequencies, and exponential tails at neither or both ends of the cline. The best model was selected by a corrected Akaike information criterion (AICc), and loci were deemed to be clinal based on those with a minimal negative log‐likelihood above the 90% quantile limit for all loci. *HZAR* provided estimates of both cline center and width for the neutral and outlier loci. Average dispersal was then calculated based on Sotka and Palumbi (2006), where dispersal is equal to the square of the cline width multiplied by a product of the maximum linkage disequilibrium (*R* ^2^) for each locus and recombination rate *r* (0.5).

### 2.5 Gene ontology

We attempted gene ontology using Blast2GO 3.3.5 (Conesa et al., 2005) on 80 bp FASTA sequences of all 41 environmentally associated outlier loci to determine whether any outlier loci were associated with genes that may be related to environmental tolerances (i.e., heatshock or immune system proteins). We applied the *blastn* algorithm and queried multiple databases including SWISSPROT and NCBI nucleotide/protein collections with an *E*‐value cutoff of 1e^−4^ and otherwise default parameters.

## 3 RESULTS

### 3.1 Environmental outlier detection

**Figure 2:**
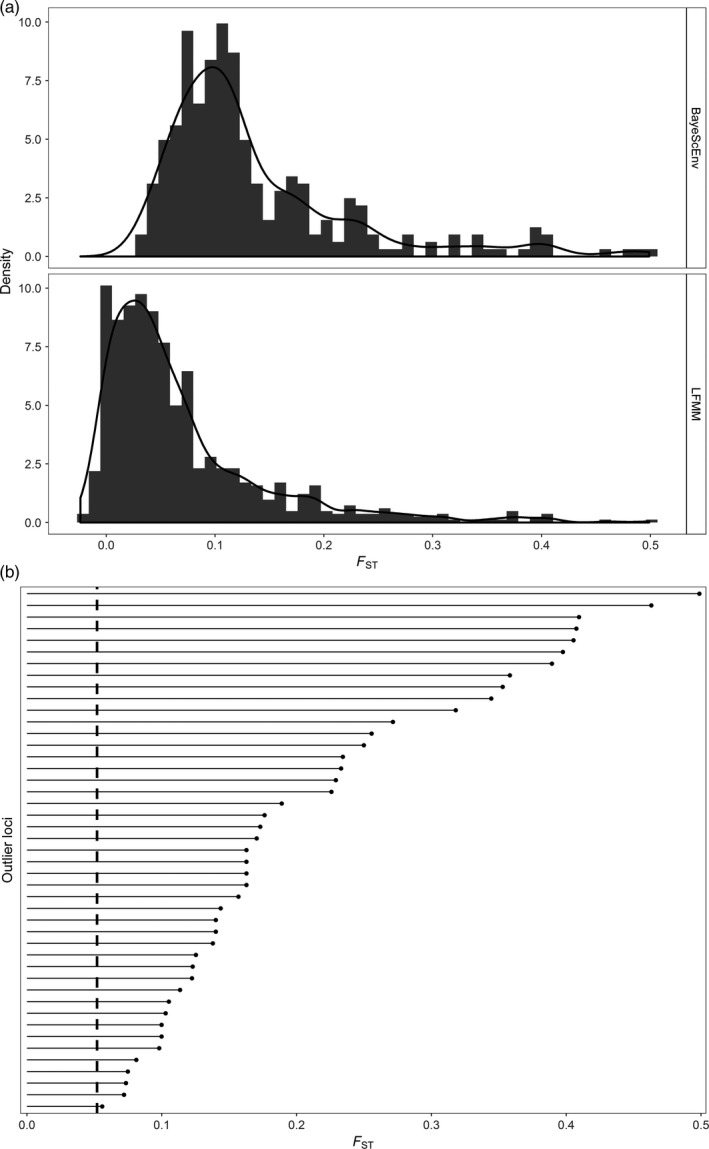
(a) *F*_ST_ frequency distributions for 302 loci detected by BayeScEnv and 770 loci detected by latent factor mixed models (LFMM) as putative outliers correlated with at least one environmental variable. (b) *F*_ST_ values for each of the 45 outlier loci that overlap between both BayeScEnv and LFMM. The dashed vertical line indicates the mean *F*_ST_ for all 9,137 loci (0.052)

**Figure 3:**
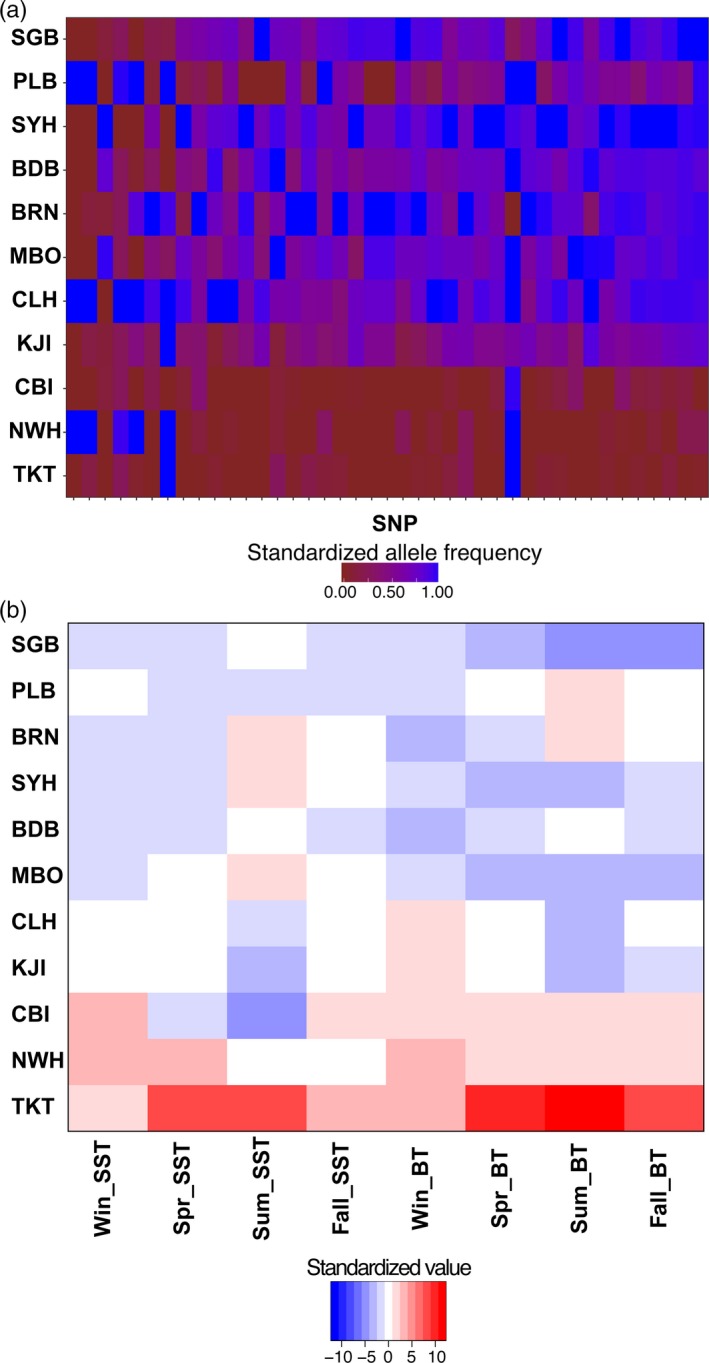
(a) Standardized allele frequency heat map for the 41 loci detected as environmental outliers. A distinct cline is observed between the southern and northern populations of green crab. (b) Heat map of site‐specific seasonal sea surface and bottom temperatures

Of the 9137 SNPs analyzed from Jeffery, DiBacco, Van Wyngaarden et al. (2017), LFMM classified 770 loci as outliers across all environmental variables at a false discovery rate of 0.05 and BayeScEnv detected 302 loci as outliers for at least one environmental variable. Of the outliers reported by LFMM and BayeScEnv, 45 loci (0.49% of all SNPs) were detected by both methods (Figure 2). We then further removed four SNPs that were located on the same RAD‐tag as other SNPs, resulting in 41 loci that were used as our environmental outlier panel, while 1,027 loci were removed from the full RAD panel to create a panel of 8,110 nonoutlier SNPs (88.76%). The outlier loci showed a clear north–south division in standardized allele frequencies (Figure 3a), which corresponds to an abrupt break in seasonal temperatures (Figure 3b). Locus‐specific *F*_ST_ values for the outlier loci ranged from 0.056 to 0.499 (mean ± *SD*: 0.23 ± 0.12; Figure 2), and linkage disequilibrium values among environmental outliers were relatively low (mean *r* ^2^ ± *SD*: 0.08 ± 0.09).

### 3.2 Principal component analysis of environmental data

**Figure 4:**
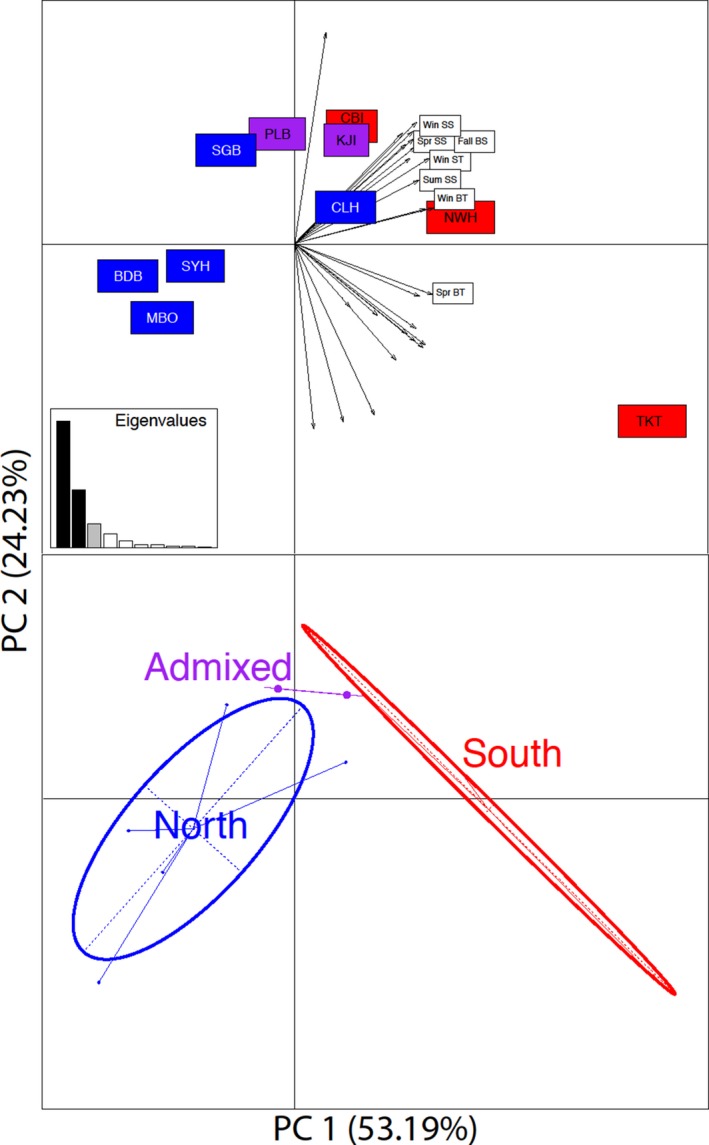
Principal component analysis on the top seven seasonal environmental variables for our 11 sampling locations based on their loadings shows a clear separation of northern and southern sites along PC1. Win, winter; Spr, spring; Sum, summer; BS, bottom salinity; BT, bottom temperature; SS, surface salinity; ST, surface temperature (top panel). Sites classified as northern, southern, or admixed, based on Jeffery, DiBacco, Wringe et al. (2017) are clearly separated based on the environment alone, with admixed sites being intermediate to the northern and southern sites (bottom panel)

A PCA of all environmental parameters at each sampling location resulted in the clustering of sampling locations that mirrored the genetically determined northern, admixed, and southern population clusters found by Jeffery, DiBacco, Wringe et al. (2017) (Figure 4). The top seven seasonal environmental variables based on loading weight on the first principal component axis were used in subsequent redundancy analysis models. A linear regression of the first principal component axis from the environmental data versus the first principal component axis from outlier allele frequency data yielded a significantly positive relationship (*r* ^2 ^= .635, *p* = .003; Figure S1), indicating a strong association between the environmental structuring and genomic structuring.

### 3.3 Environmental associations with genotype

**Figure 5:**
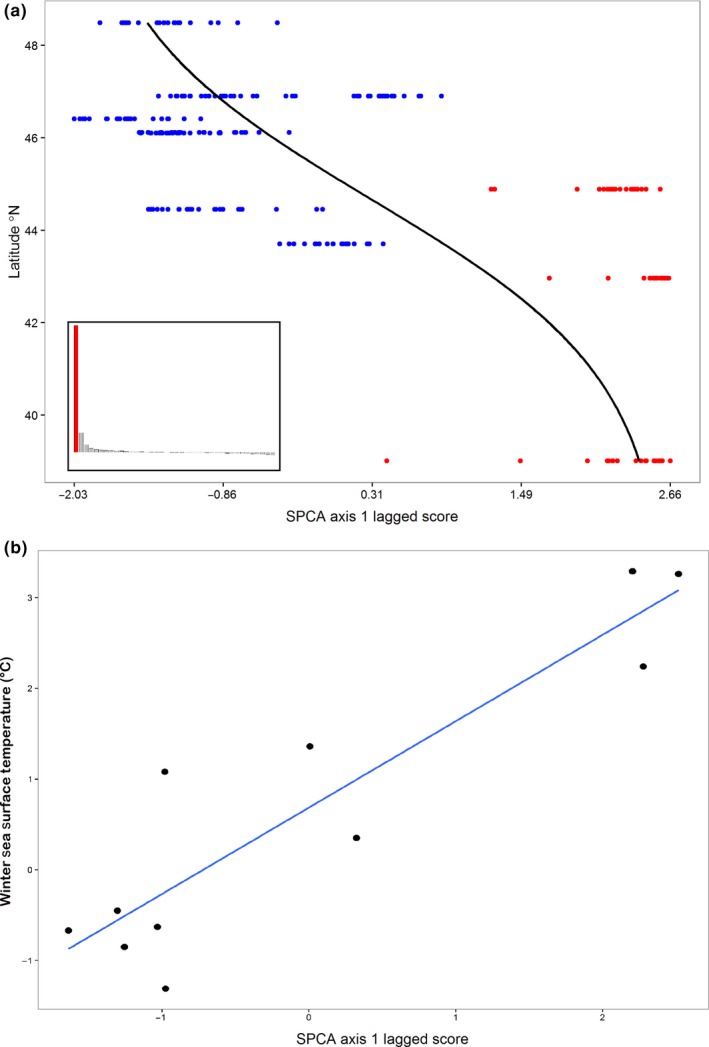
(a) The observed logistic relationship between the lagged scores of a spatial principal component analysis for our environmental outlier loci and latitude, showing a transition from the southern to northern zones at approximately 44°N. The inset shows the spatial principal component analysis (SPCA) eigenvalues showing a high degree of global structure (extreme positive eigenvalue) but little local structure (negative eigenvalues). (b) A significant relationship is observed (*r* ^2 ^= .90, *p* = .04) between the axis 1 lagged scores from a spatial principal component analysis on environmental outlier genotypes and winter sea surface temperature

Separate SPCAs on both the environmental outlier and nonoutlier datasets revealed a significant degree of global structure (*p* = .001) and little local structure (*p* = 1.0) as noted by the extreme positive eigenvalue that explained 66% of the observed structure (Figure 5a inset). A logistic regression of SPCA lagged scores against latitude showed a clear cline in population structure (Figure 5a). A multiple linear regression of the SPCA axis 1 lagged scores for the outlier loci against each seasonal environmental predictor with a VIF <5 found that both winter sea surface temperature (WinSST $R_{\mathrm{adj}}^{2}$  = .90, *p* = .002; Figure 5b) and summer bottom temperature (*p* = .04) were significantly associated (Table S1). Similarly, elastic net regression selected a model with only two predictors—WinSST and SumBT (coefficients 0.64 and 0.08, respectively, all others = 0)—that best correlated with the outlier and nonoutlier SPCA lagged scores.

**Figure 6:**
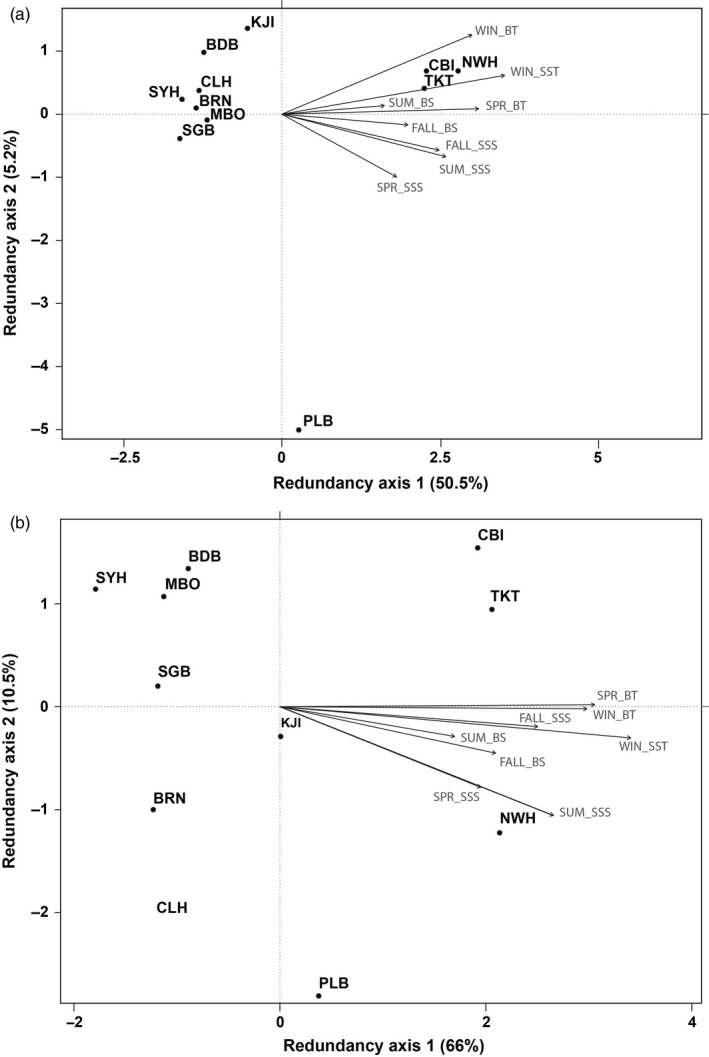
(a) A redundancy analysis on the seasonal temperature environmental variables and neutral loci allele frequencies suggests winter sea surface temperature as the most significant predictor of genetic differentiation. (b) Redundancy analysis on the outlier SNPs additionally suggests winter sea surface temperature as the most significant predictor genetic differentiation

The relationship between WinSST and observed population‐genetic structure was also highlighted by the redundancy analysis, which revealed WinSST to be the most significant environmental variable associated with the outlier (*F *=* *13.03, *p* = .005) and nonoutlier SNP genotype distribution (*F *=* *4.5024, *p* = .02) selected by the *ordistep* function (Figure 6). When accounting for geographic proximity among sites using a partial RDA, WinSST was again the top predictor of both outlier and nonoutlier genotypes based on model selection, although this was marginally nonsignificant for the nonoutlier SNPs (*F *=* *13.03, *p* = .005 and *F *=* *2.41, *p* = .055, respectively). Subsequent analyses of variance revealed that WinSST was the only significant predictor of both the nonoutlier and outlier variation (*F *=* *11.80, *p* = .002 and *F *=* *10.45, *p* = .007, respectively). Variance partitioning based on the outlier RDA models revealed that the environment explains 43.95% and geography explains 9.35% of the variation in genetic structure, with the remaining variance explained by the interaction between environment and geography.

Using multiple regression on distance matrices, we observed a significant isolation‐by‐distance relationship among populations (*r* ^2 ^= .39, *p* = .004) largely driven by inter‐regional comparisons. However, an MRM model including winter sea surface temperature had a stronger relationship with pairwise *F*_ST_ values (*r* ^2 ^= .55, *p* = .002) suggestive of this environmental variable's role in possibly constraining gene flow.

### 3.4 Estimation of dispersal

Twenty‐four of 41 outliers (58.5%) were considered clinal based on their log‐likelihood distribution, while only 10 of the randomly selected 100 nonoutlier loci (10%) were considered clinal (Figure S2). The center of the cline was estimated to be 994.91 ± 291.67 km (mean ± *SD*) from the southernmost sites, while the cline width was estimated to be 299.22 ± 666.88 km based on outlier allele frequencies. The 10 clinal nonoutlier loci estimated the cline center at 955 ± 145.83 km and a cline width of 477.07 ± 793.07 km. Neither the cline centers (*p* = .62) or the cline widths (*p* = .56) were significantly different between nonoutlier and outlier loci when correcting for sample size. Nonoutlier loci estimated the standard deviation of parent‐offspring distance to be 82.31 ± 127.71 km (mean ± *SD*). The standard deviation of parent‐offspring distance was estimated to be 138.50 ± 319.95 km based on the clinal environmental outliers.

### 3.5 Gene ontology

Our Blast2GO search of 41 outlier loci yielded only one reliable result, which aligned an outlier sequence to a Toll‐like receptor (TLR3) from the decapod *Portunus trituberculatus* at an e‐value of 1.6e^−20^. TLRs are cell‐surface receptors which aid in the innate immune system (Li, Li, Wang, & Song, 2015) and as such may have variable alleles that are advantageous in different environments.

## 4 DISCUSSION

Here, we present results consistent with the environmentally associated distribution of secondary contact punctuated by an environmental gradient among two independent invasions of green crab along the coast of eastern North America. Our multivariate analyses reveal winter sea surface temperature to be the best predictor of spatial structure after accounting for multicollinearity among environmental predictors. Similarly, multiple regression on distance matrices revealed a stronger isolation‐by‐environment relationship than a simple isolation‐by‐distance relationship, suggesting that the environment is more of a constraining influence on gene flow than geographic distance among populations (Keller, Van Strien, & Holderegger, 2012). Our results show that in the zone of secondary contact, an abrupt change in winter surface temperature, and to a lesser degree, summer bottom temperature, correlates with a nonlinear shift in allele frequency in both the outlier loci and the nonoutlier loci between green crab ecotypes. These variables represent seasonal extremes (lows and highs), which intersect with the physiological tolerances of each ecotype carried over from their native range, and are likely factors which limit gene flow and range expansion in this species (Tepolt & Somero, 2014). In the native range, green crab show a genetic continuum of mitochondrial lineages along their latitudinal gradient (Roman & Palumbi, 2004) and show large differences in structure between northern and southern populations (Tepolt & Palumbi, 2015) entirely consistent with the patterns observed here. The broad invasive geographic range of green crab in eastern North America was likely made possible by the two independent invasions originating from very different thermal regimes in the native range, a fact that is supported by evidence that the initial southern invasion stalled in the Halifax region (~44.5°N) in the 1960s, perhaps limited by the cold seasonal temperatures north of this region. This system is unique in this respect, where two genetically distinct lineages underwent independent invasions, allowing for a broad colonization and the study of environmental associations in a newly invasive and highly dynamic system.

This works build directly on a large body of literature on secondary contact both in native and among native and invasive species (Fitzpatrick et al., 2010; Saarman & Pogson, 2015; Viard et al., 2016). For example, in the *Mytilus* mussel complex, three species exhibit strong barriers to reproduction, although hybridization still occurs (Saarman & Pogson, 2015). A hybrid zone exists between the invasive *M. galloprovincialis* and native *M. trossulus* in California, a system which has existed for several hundred years; Saarman and Pogson (2015) suggest that the transfer of adaptive genes from *M. edulis* to *M. galloprovincialis* in the species’ native European range may have contributed to the invasive success of *M. galloprovincialis*. Similarly, Barred Tiger salamanders (*Ambystoma tigrinum mavortium*) recently introduced (<60 years) into the native habitat of the California Tiger salamander (*A. californiense*), form a strong hybrid zone with F_1_ hybrids showing increased fitness relative to either pure parent species (Fitzpatrick et al., 2010). However, unlike in species which show both endogenous and exogenous barriers to gene flow (Saarman & Pogson, 2015), green crab ecotypes interbreed successfully (Jeffery, DiBacco, Wringe et al., 2017), although regions of secondary contact are environmentally intermediate relative to the habitat of northern or southern ecotypes. Collectively, these studies reinforce the need for knowledge of the demographic history and proper interpretation of the adaptive or neutral processes that lead to the creation of hybrid zones, particularly in recent introductions which may have not yet reached equilibrium.

### 4.1 Seascape genomics of marine animals

Studies that directly relate environmental data to population‐level genomic data in aquatic species (“seascape genomics”) are rare, and to our knowledge, no studies exist that integrate genomic data and fine‐scale environmental data in an invasive species with multiple invasions (Sherman et al., 2016). Recent investigations of seascape genomics for North American Atlantic species have detected significant associations between the environment and structure in Atlantic cod (*Gadus morhua*) (Bradbury et al., 2010), American lobster (*Homarus americanus*) (Benestan et al., 2016), and sea scallop (*Placopecten magellanicus*) (Van Wyngaarden et al., 2017). Sea bottom temperature was revealed to be correlated with outlier loci on both sides of the Atlantic within cod, suggestive of parallel evolution in distinct cod lineages (Bradbury et al., 2010). For American lobster, Benestan et al. (2016) revealed that ocean currents best explained neutral population structure, but that minimum annual sea surface temperature (SST) was likely driving local adaptation. Because adult lobsters are benthic, it was hypothesized that this minimum SST is experienced by pelagic larval lobsters directly affects their mortality, thus leading to local adaptation in warmer or colder regions. Van Wyngaarden et al. (2017) concluded that cold temperatures, including minimum annual and winter temperatures, in addition to differences in salinities among sites, potentially restrict gene flow among northern and southern populations, leading to strong latitudinal structuring in this species. Similar to green crab, the native cod, scallop, and lobster show strong north–south clustering in the Northwest Atlantic, and are primarily structured by periods of extreme cold in the winter which limits gene flow between northern and southern populations and may impose a selective pressure. Whereas both American lobster and sea scallop are native to this region, it seems reasonable to hypothesize that invasive green crab are likely adapted to different temperatures from their native range and that this may be limiting range expansion and secondary contact in eastern North America (Tepolt & Somero, 2014).

### 4.2 Green crab environmental limits

Our results suggest that winter sea surface temperature primarily maintains the spatial structure of green crab ecotypes in eastern North America. It is this cold seasonal temperature that likely constrains the range and gene flow of two genetically distinct invasions, showing an abrupt break in population structure within their northwestern Atlantic range, except for a zone of secondary contact in southern Nova Scotia. The broad‐scale environmental correlations observed in this study are most likely carried over from structure in the native range, as both our outlier and nonoutlier loci showed similar relationships to temperature, potentially reflective of the long‐term divergence between ecotypes in Europe (Jeffery, DiBacco, Wringe et al., 2017). Because the observed environmental associations are present both in outlier and nonoutlier loci (although to a lesser extent), a simplistic interpretation of the factors responsible for these associations remains elusive. The environmental associations observed within outlier loci may represent a genomewide signal that is the result of several historical processes, including selection, drift, or both, in the native range, rather than true genomic islands of divergence that are associated with temperature‐dependent selection. These loci may also represent alleles that are intrinsically incompatible between ecotypes that have become associated with the environment along a climatic gradient via the endogenous–exogenous barriers “coupling hypothesis” (sensu Bierne et al., 2011). Without genomic data from the native range and a comprehensive green crab genome, these hypotheses cannot be tested directly here. Interestingly, some outlier loci in NWH, PLB, and CLH showed the opposite pattern of allele frequency than is expected based on the majority of outliers. Although these loci showed no obvious trend with environmental variables, this pattern suggests the presence of genomic heterogeneity within the green crab genome and variation in evolutionary processes involved, such as selection or introgression between ecotypes across the genome.

WinSST represents the extreme low temperatures these crabs face annually, although it remains to be seen which life stages (postsettled juveniles or adults) are most affected by this minimum temperature. Reproductive timing varies across the invasive range, with mating typically occurring in the summer or early fall (Best, McKenzie, & Couturier, 2017; Klassen & Locke, 2007), and as larvae spend several weeks suspended in the water column, it may be that postsettled juveniles of southern ecotype crabs are unable to survive the decrease in sea surface temperature in the northern region (deRivera et al., 2007). Our finding that WinSST had the most robust relationship with ecotype distributions is consistent with the known environmental tolerances of adult green crab and their ability to grow and survive at different latitudes (Lowen, McKindsey, Therriault, & DiBacco, 2016). Additionally, previous studies have noted that cold winter water temperatures are linked to higher adult mortality and lower recruitment (Berrill, 1982; Beukema, 1991), while warmer winters may be linked to stronger year‐classes in green crab populations in western North America (Yamada & Kosro, 2010). A recent review of environmental factors influencing the local (<10 km) green crab population structure suggests that water depth and biotic interactions were the primary variables influencing distribution (Cosham, Beazley, & McCarthy, 2016). Interestingly, Cosham et al. (2016) found that temperature did not directly influence local distribution of green crab across its native European range, although this was only at the fine‐scale within regions and ultimately not at the scale where the variation in temperature might evoke a selective pressure or impose significant constraints on gene flow.

Our principal component analysis on the environmental data showed distinct northern and southern groupings that correlate strongly with previously detected genetic spatial structure. Known hybrid sites—Kejimkujik, NS and Placentia Bay, NL—appear intermediate to the northern and southern sites in both the environmental and genotypic PCAs (Blakeslee et al., 2010; Jeffery, DiBacco, Wringe et al., 2017). This suggests that both northern and southern ecotypes are able to interbreed in these zones of secondary contact, although the physiological tolerances of hybrid individuals require testing to determine why hybrids are not found in high abundance in other parts of their invasive range (Jeffery, DiBacco, Wringe et al., 2017). Despite its northern location, our PCA suggests that Placentia Bay is similar to Kejimkujik in terms of its environment, and indeed Placentia Bay has warmer surface waters than most of southern Newfoundland, but is still colder than the Maritimes overall (Craig & Colbourne, 2004). The hybrid crabs’ ability to survive in this environment likely depends on their ability to move deeper into the bay to avoid the cold winter surface temperatures, which they cannot do in the more exposed St. George's Bay region of Newfoundland. Similar results of interspecific hybridization in anemonefish in the Arabian Peninsula showed an admixture zone in a region associated with intermediate environmental conditions, suggesting differential local adaptation and intermediate physiological tolerances in hybrid individuals (Saenz‐Agudelo et al., 2015). However, it remains to be seen whether hybrid green crabs have a greater or intermediate environmental tolerance relative to the pure northern or southern individuals, as the successful establishment of hybrid populations requires that hybrids persist in either intermediate or broader niches relative to their parental populations or species to avoid being outcompeted (Pruvost, Hollinger, & Reyer, 2013; Seehausen, 2004).

Collectively as a species, green crab have a wide tolerance of a range of temperatures and salinities (Klassen & Locke, 2007). Their broad thermal tolerance, however, is partitioned into the separate ecotypes when the underlying genetic structure is taken into consideration (Tepolt & Somero, 2014). In both their native and introduced ranges, southern populations of green crab have a higher cardiac critical temperature before heart failure than northern populations, while northern populations had a lower critical temperature before heart failure, indicative of potential temperature adaptation and/or acclimatory plasticity (Tepolt & Somero, 2014). Differences in thermal tolerance found in the eastern North America invasive range are likely carried over through their initial introduction, leading to the observed clinal structure of this species. Tepolt and Somero (2014) admit that their study cannot differentiate plasticity from long‐term adaptation, but that both processes have likely played a role in the global invasions of green crab. While cardiac critical temperature is just an acute metric of thermal tolerance, these physiological differences corroborate our overall result that winter sea surface temperature is an important factor influencing population structure and limiting gene flow of northern and southern ecotypes in eastern North America, which has been maintained since the separate introductions of these ecotypes from their native range.

### 4.3 Clines and estimates of dispersal

Estimating larval dispersal rates has historically been achieved using oceanographic models and physical or experimental observation of propagule movement, as well as field observations of the rate of spread of invasive species (Selkoe & Toonen, 2011; Shanks, Grantham, & Carr, 2003). We used a model of cline width calculated from allele frequencies proposed by Sotka and Palumbi (2006) and based on clinal models of gene flow (Barton & Gale, 1993) to estimate per‐generation dispersal in eastern North America. Our approximations of the center of clines in allele frequency using both putative outliers and nonoutliers was consistent with the “KJI” sample location in southeastern coastal Nova Scotia, a known zone of secondary contact (Blakeslee et al., 2010; Darling, Bagley, Roman, Tepolt, & Geller, 2008; Jeffery, DiBacco, Wringe et al., 2017). Nonoutlier and outlier clinal loci yielded estimates of dispersal on similar orders of magnitude (~80–140 km/generation) and are similar to estimates reported by Grosholz (1996); Klassen and Locke (2007) and Shanks et al. (2003) for green crab, and are consistent with the high degree of genomewide divergence detected between ecotypes (Jeffery, DiBacco, Van Wyngaarden et al., 2017).

The same model was recently implemented by Van Wyngaarden et al. (2017) to study effective dispersal in the sea scallop (*P. magellanicus*) along a similar range in eastern North America. However, this clinal model assumes balance between natural selection and dispersal rate, which may not be at equilibrium in the highly dynamic green crab system. Similarly, changes in linkage disequilibrium and allele frequencies over time will inevitably alter the estimated cline width, and therefore, we consider our model an approximation of contemporary rates of dispersal.

The single clinal outlier locus that aligned to a reference sequence corresponded to a Toll‐like receptor (TLR) gene which is part of the innate immune system (Li et al., 2015). While speculative, the fact that this locus correlates with the environment suggests a potential relationship between different TLR alleles and latitude in green crabs, possibly due to differences in marine parasite species richness at different latitudes (Rohde, 2005; Rohde & Heap, 1998). For example, unique and highly conserved TLRs were revealed within the Atlantic cod genome (Star et al., 2011), a species which has also been shown to show genomic differences associated with temperature across a latitudinal range (Bradbury et al., 2010). Marine pathogens are sensitive to temperature, and disease impacts are predicted to increase in severity as the ocean warms (Harvell et al., 2002). As such, aquatic species with latitudinal variation in alleles associated with immune function, such as we suggest for green crab and has been shown for Atlantic salmon (Dionne, Miller, Dodson, Caron, & Bernatchez, 2007), may show different responses to a changing climate. Without data from the crabs’ native range and a comprehensive genome sequence, we cannot determine whether this outlier‐associated locus is the result of balancing or divergent selection, a possible adaptation carried over from the crabs’ native range, or perhaps the result of a transient epizootic event leading to intermittent selection for alleles of this locus which happen to correspond to different environments (Agrawal, 2011). The fact that only one match was detected by Blast2GO suggests a lack of genomic resources available within crustaceans, or perhaps the complex demographic history of green crab complicates the identification of genomic regions currently experiencing selection, and could result in poor initial outlier detection. Nevertheless, additional genomic data from green crab and other decapods would undoubtedly assist in understanding the genes and genomic architecture involved in the delineation of the two invasive ecotypes.

### 4.4 Implications

Understanding the broad‐ and fine‐scale environmental factors that influence an invasive species’ distribution on a temporal scale and at different life stages is necessary to model the range expansion and ecological or economic impacts of that species over time (Cosham et al., 2016). Overall, our results suggest that winter sea surface temperature could be an important environmental variable to predict any future expansion of green crab in eastern North America. While the southern ecotype of green crab is largely limited to south of and including the Bay of Fundy, climatic predictions of warming sea surface temperatures in the region could allow for displacement of the northern ecotype. At the same time, the northern ecotype is currently found as far north as the north coast of Newfoundland, and further northward spread is either limited by dispersal ability, including less frequent anthropogenic transport north of Newfoundland and the natural southwestern current flow (Pringle, Blakeslee, Byers, & Roman, 2011), or sea surface temperature. To date, green crab have been treated as a single population or lineage within Canada for monitoring strategies (Therriault, Herborg, Locke, & McKindsey, 2008), but evidence here suggests that different ecotypes should be modeled separately to determine potential for range expansion and impacts on coastal ecosystems. While this study only considered annual and seasonal temperature and salinity, other factors including ocean currents (e.g., Benestan et al., 2016), water depth, and fine‐scale biotic interactions (Cosham et al., 2016; Rossong et al., 2012) could influence selection but were not considered as the focus of this study was on the factors that may influence broad‐scale population structuring of northern and southern ecotypes.

### 4.5 Limitations

Although we were able to detect genetic‐environment associations between a small proportion of outlier loci and season temperatures, we cannot conclude which processes have led to this observation without data from the crabs’ native range, a well‐annotated genome, and a comprehensive understanding of the temporal dynamics of the green crab system. As mentioned, it is likely green crab in their native range are adapted to a range of ocean temperatures from North Africa to Norway, and may show both genetic variation and plasticity in thermal tolerances. The recent introductions of different lineages to North America, and even more recent secondary contact between lineages, appear to simply reflect the native population structure, and a clinal gradient in structure is maintained by a corresponding gradient in temperature, in particular winter bottom temperature. Data from populations in the native range at different latitudes could aid in determining how seasonal minima, such as winter bottom temperature, might also constrain population structure in a naturally replicated system (see Schmidt et al., 2008). Finally, additional genomic resources from green crab, other decapods, or crustaceans in general, including annotated genomes or transcriptomic data, could lead to the identification of other gene regions that show relationships between allele frequency and environment, shedding light on the processes which have led to this observed population structure and any reproductive barriers associated with constraints in gene flow.

### 4.6 Summary

The population structure of two green crab ecotypes and hybrid populations in the Northwest Atlantic Ocean is driven on a broad scale by a gradient in seasonal temperature minima, leading to reduced gene flow and constraints in the ranges of two distinct ecotypes which evolved along a latitudinal gradient in Europe. Winter sea surface temperature was observed to show the strongest relationship with crab genetic spatial structure, suggesting that the continued expansion of the separate ecotypes is limited by the minimum temperature each year. This broad understanding of how the environment limits the genetic spatial structure of invasive green crab can be implemented in models predicting the spread and impacts of this species over time, and allow for projections of range expansion under climate change scenarios.

## DATA ARCHIVING

Data for this study are available from the Dryad Digital Repository: https://doi.org/10.5061/dryad.bq27d.

## Supporting information


** **
