# Supplementary material for: Genomewide evidence of environmentally mediated secondary contact of European green crab (Carcinus maenas) lineages in eastern North America
Source: Evol Appl. 2018 Feb 23;11(6):869–82. doi: 10.1111/eva.12601 (PMC5999199; doi:10.1111/eva.12601)
Supplement: Supplementary file 1 [file EVA-11-869-s001.docx]

**Supplementary Tables and Figures**

Supplementary Table 1. Results from a multiple linear regression on four environmental variables which had a variance inflation factor <5. Winter sea surface temperature and summer bottom temperature were the only variables that significantly correlate with the axis 1 lagged scores from a spatial principal components analysis on outlier SNP genotypes.

| **Environmental Variable** | **Estimate** | **Std. Error** | **t-value** | **P-value** |
| --- | --- | --- | --- | --- |
| (Intercept) | -0.216 | 1.209 | -0.179 | 0.864 |
| SeasonalSurface_win_sst* | 0.846 | 0.165 | 5.117 | 0.002 |
| SeasonalBottom_bt_sum | 0.142 | 0.055 | 2.556 | 0.043 |
| SeasonalSurface_fall_sst | -0.230 | 0.179 | -1.281 | 0.247 |
| SeasonalSurface_sum_sst | 0.022 | 0.078 | 0.289 | 0.782 |


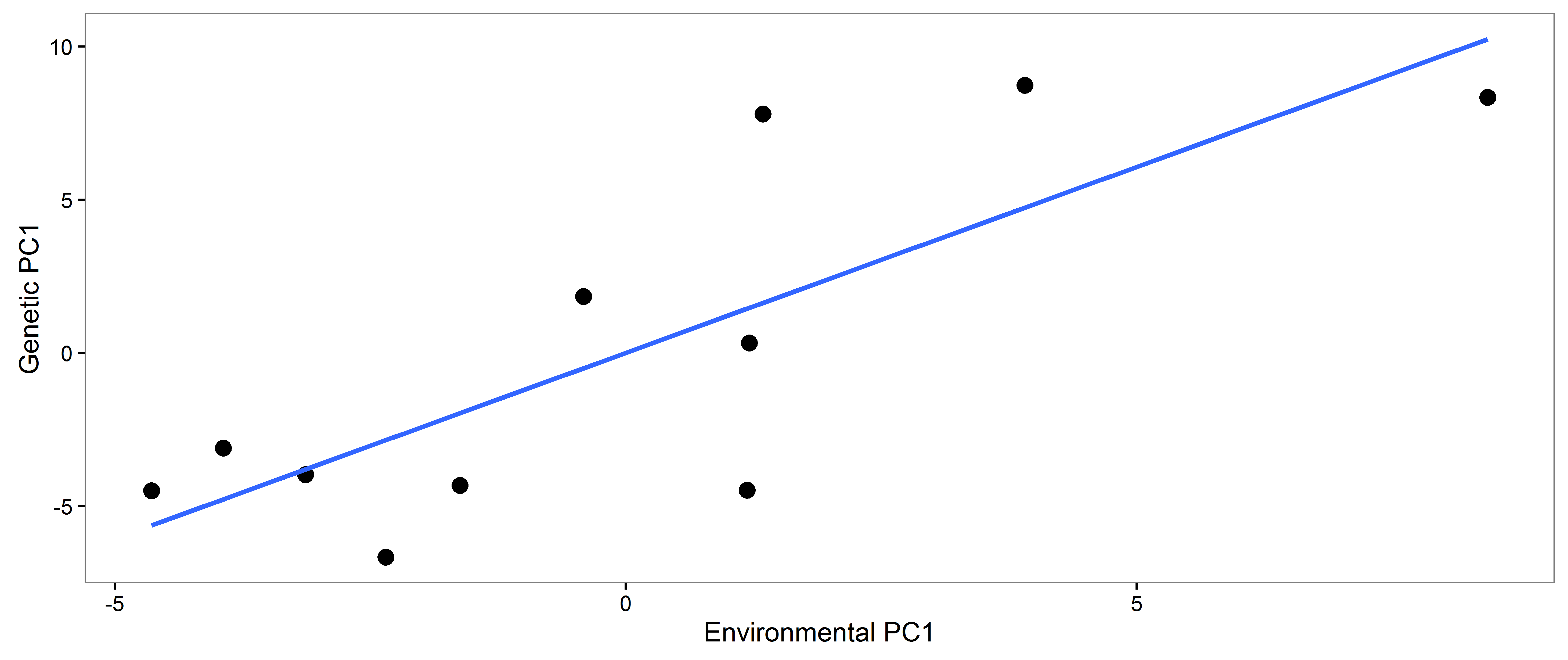


Figure S1. A linear regression of the first principal component axis from the environmental PCA versus the first axis from the genetic PCA reveals a significant positive relationship (r^2^=0.635, p=0.003).


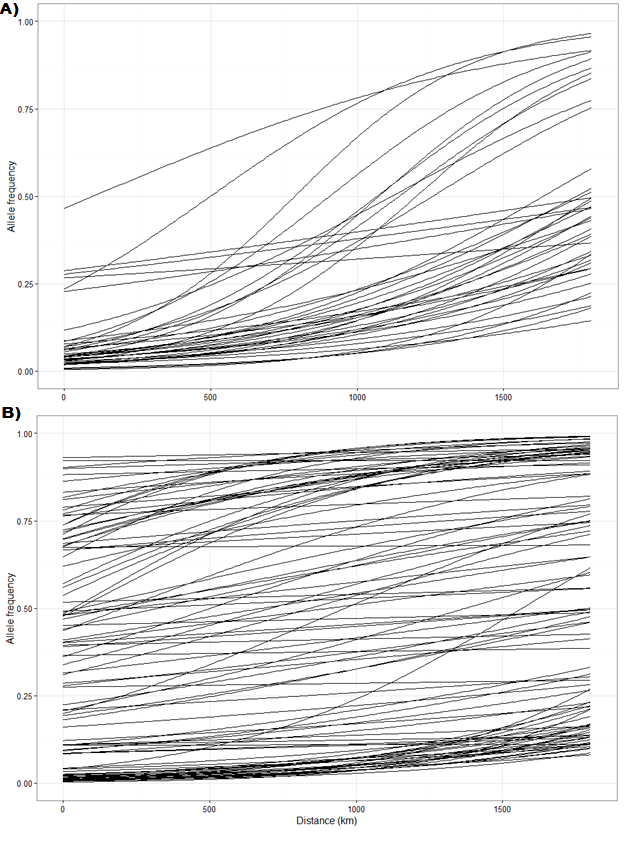


Figure S2. Plots of allele frequency against least-cost distances among 10 sampling sites for A) 41 environmental outlier loci and B) 100 randomly selected non-outlier loci.
